# Supplementary material for: Self-cleaving guide RNAs enable pharmacological selection of precise gene editing events in vivo
Source: Nat Commun. 2022 Nov 30;13:7391. doi: 10.1038/s41467-022-35097-5 (PMC9712609; doi:10.1038/s41467-022-35097-5)
Supplement: Supplementary file 1 — Supplementary Information [file 41467_2022_35097_MOESM1_ESM.pdf]

# Self-cleaving guide RNAs enable pharmacological selection of precise gene editing events in vivo

## Supplementary Information

# Supplementary Information

### Supplemental Figure 1

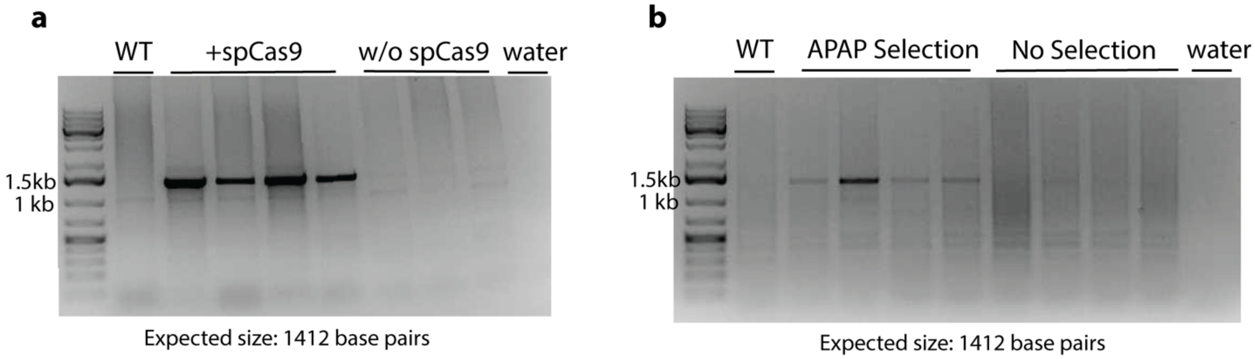


**Supplemental Figure 1: Integration into the Album locus.** Agarose gel of PCR products to confirm integration into the Albumin locus in (a) mice that received the GR-Hpd scgRNA rAAV (+spCas9 n=6; w/o SpCas9 n=4) and (b) mice that received the GR-Cypor scgRNA rAAV (APAP selection n=4; No selection n=4). Primer pairs were designed with one primer outside of the arm of homology and one primer in the P2A transgene.

**Supplemental Figure 2**

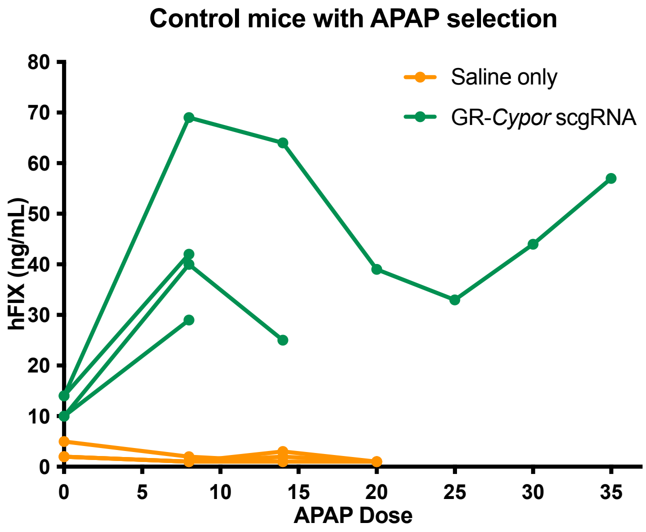


**Supplemental Figure 2**: **Human F9 levels in control mice.** Circulating human F9 concentrations in mice that received either saline only or the GR-*Cypor* scgRNA without spCas9 that were treated with APAP following the same schedule as experimental mice in Figure 2. Each line represents one mouse. Lines terminate when the animal was found deceased or required euthanasia. Source data are provided as a Source Data file.

**Supplemental Figure 3**

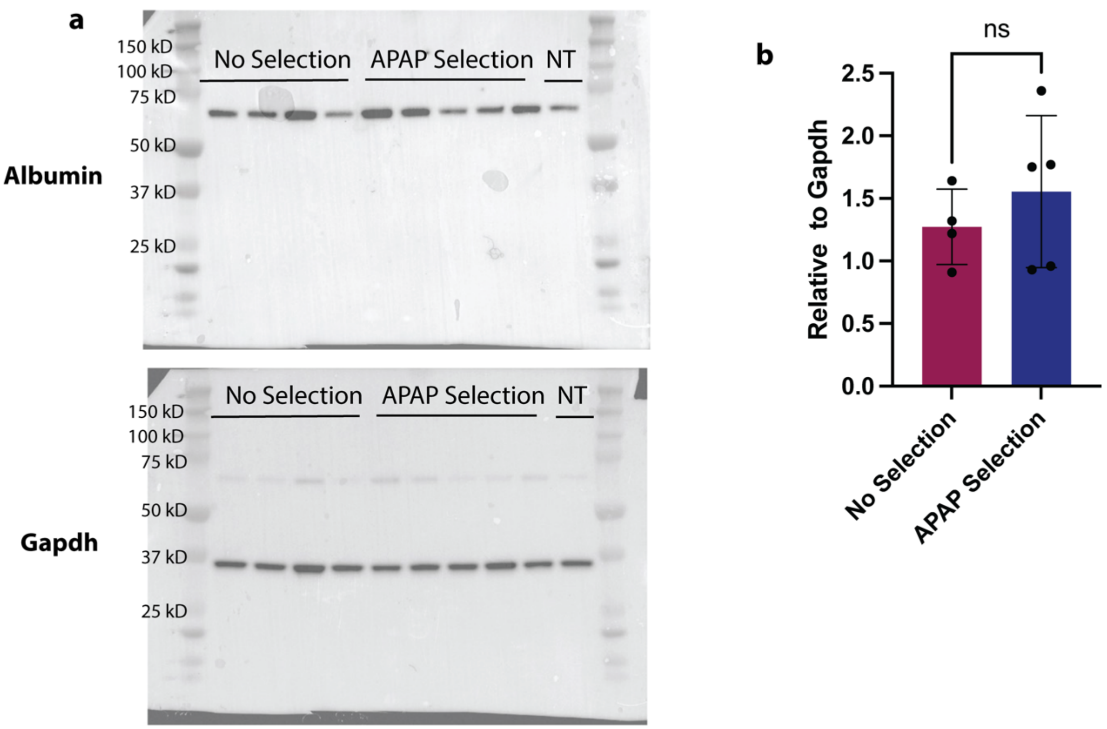


c

| Location | Tags Per Million (TPM) Hpd-miR | Unselected (Cypor scgRNA) | Selected (Cypor scgRNA) | Ratio of selected/unselected |
|---|---|---|---|---|
| Alb | 16542 ± 5853 | 32459 ± 12647 | 33670 ± 6653 | 1.04 |
| Human F9 | 1909 ± 545 | 7.78 ± 6.07 | 394.8 ± 136.8 | 50.7 |
| Cypor | 267 ± 43.4 | 345.0 ± 85.6 | 331.8 ± 44.7 | 0.96 |
| Ttr | 7919 ± 1412 | 4573 ± 620.8 | 3908 ± 548.8 | 0.85 |
| Aat | 1129 ± 597 | 4170 ± 270.6 | 4652 ± 142.8 | 1.12 |
| Tbp | 13.0 ± 1.68 | 7.24 ± 2.16 | 8.18 ± 0.30 | 1.13 |
| Gapdh | 1531 ± 496 | 977.2 ± 57.8 | 948.1 ± 82.7 | 0.97 |

d

| Location | Relative to β-actin Hpd-miR |
|---|---|
| Alb | 2.19 ± 0.929 |
| Human F9 | 5.22 ± 1.48 |
| P2A | 0.217 ± 0.079 |

**Supplemental Figure 3: Protein and RNA analysis** (a) Western blot for Albumin and Gapdh using total protein collected from liver homogenate of mice that received the GR-Cypor-scgRNA rAAV that underwent no selection or APAP selection. NT is from a mouse that received no treatment. The faint upper band in the Gapdh blot is the result of incomplete striping of the blot prior to reprobing with the anti-Gapdh antibody. (b) Quantification of the abundance of Albumin protein relative to Gapdh. Data are presented as mean values and error bars represent standard deviation. Statistics were calculated using a two-sided unpaired t test. No selection n= 4 animals; APAP selection n= 5 animals. Source data are provided as a Source Data file. (c) Table of tags per million (TPM) of hepatocyte-specific and housekeeping genes of interest from RNA sequencing. Hpd-miR mice received the original GeneRide vector containing a selectable Hpd shRNA[1]. (d) Table showing expression of Albumin, human F9 and P2A relative to beta-actin in mice that had received the original GeneRide vector containing a selectable Hpd shRNA[1].

**Supplemental Figure 4**

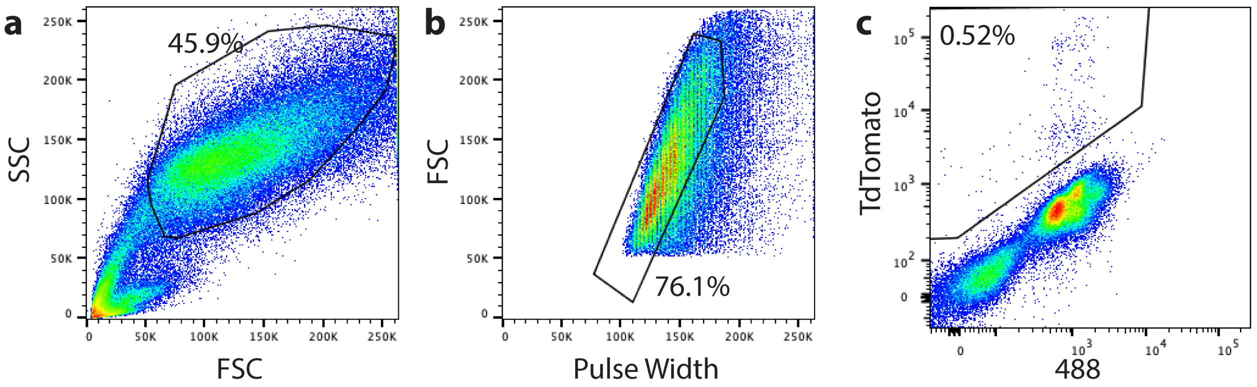


**Supplemental Figure 4: Gating Strategy for Flow Cytometry.** Sequential gating was used to (a) exclude non-cell events, (b) then exclude doublets and larger cell clusters then (c) gate for tdTomato positive hepatocytes. FSC= Forward scatter, SSC = Side scatter

## Supplemental Figure 5

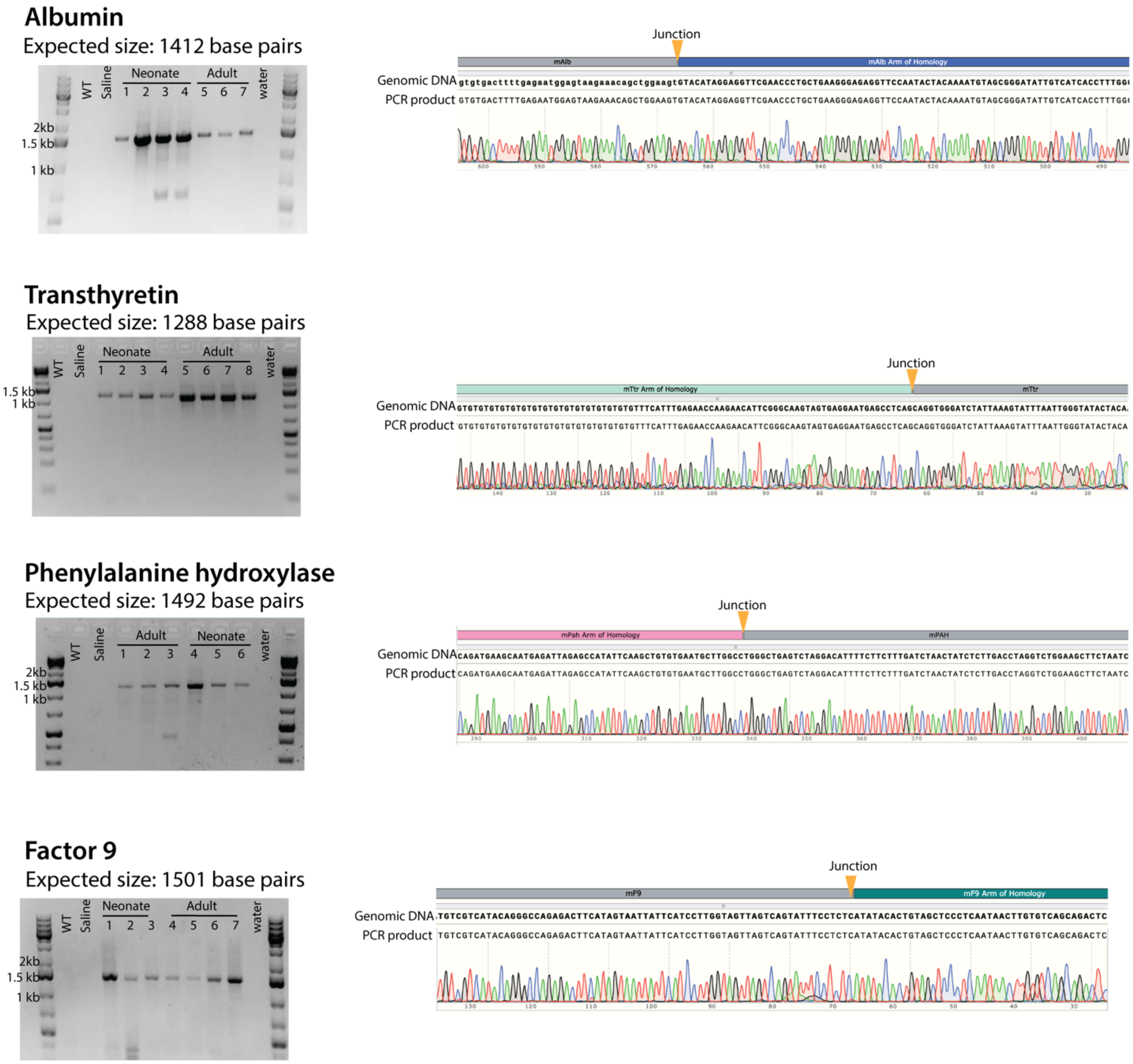


**Supplemental Figure 5: Integration of the GeneRide vector into lower expressing loci.** Agarose gel of PCR products and Sanger sequencing to show integration of the GR-stop scgRNA vectors targeting weaker expressing hepatocyte specific genes. PCRs were performed to confirm integration by designing primers pairs with one primer outside of the arm of homology and one primer in the hF9 transgene. A chromatogram for each of the targeting loci shows the location of the junction between the homology arm and endogenous DNA sequence to demonstrate integration of the GeneRide vector. (Albumin: neonate n=4, adult=3; Transthyretin: neonate=4, adult=4; Phenylalanine hydrolase: neonate n=3, adult n=3; Factor 9: neonate n=3; adult n=4).

**Supplemental Figure 6**

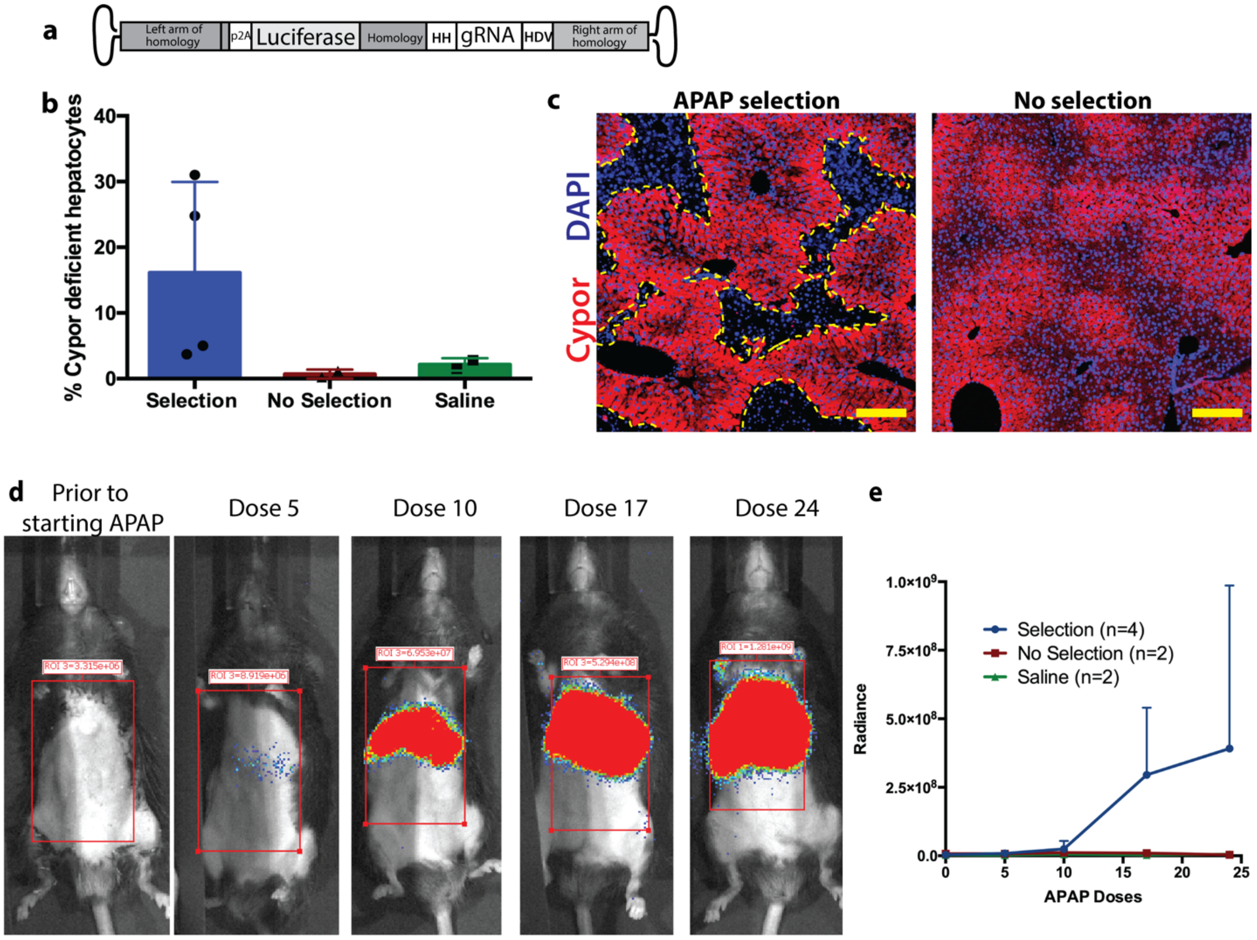


**Supplemental Figure 6: Selection in the lower expressing Transthyretin (Ttr) locus** (a) Schematic of the rAAV vector. The rAAV contains arms of homology to TTR. The arms of homology flank a P2A, a luciferase expression cassette and a scgRNA targeting Cypor. (b) Quantification of Cypor insertion and deletion frequency following terminal harvest in mice that received the AAV-*Cypor* scgRNA-luciferase that underwent APAP selection or no selection and mice that received a saline vehicle with APAP selection. Selection n= 4 animals; No selection n=2 animals; Saline n=2 animals. (c) Representative immunofluorescence staining against Cypor in a liver section from an APAP selected and a no selection mouse. Scale bar represents 200 µm. (d) In vivo (IVIS) imaging of luminescence in an animal prior to APAP selection and following doses 5, 10, 17 and 24 of APAP. (e) Quantification of luciferase expression during APAP selection. Data are presented as mean values and error bars represent standard deviation. Source data are provided as a Source Data file.

**Supplemental Table 1. Quantification of hF9 by image analysis.** Approximate quantification of hF9 positive liver area by measurement of representative immunofluorescent images.

| GR-*Hpd* scgRNA | |
|---|---|
| Mouse ID | % hF9 positive hepatocytes |
| A | 71.3% ± 14.5% |
| B | 84.0% ± 9.9% |
| C | 69.3% ± 5.9% |

| GR-*Cypor* scgRNA | |
|---|---|
| Mouse ID | % hF9 positive hepatocytes |
| A | 16.6% ± 13.3% |
| B | 15.3% ± 11.9% |
| C | 14.0% ± 9.6% |
| D | 22.7% ± 16.1% |
