# Supplementary material for: Self-cleaving guide RNAs enable pharmacological selection of precise gene editing events in vivo
Source: Nat Commun. 2022 Nov 30;13:7391. doi: 10.1038/s41467-022-35097-5 (PMC9712609; doi:10.1038/s41467-022-35097-5)
Supplement: Supplementary file 3 — Reporting Summary [file 41467_2022_35097_MOESM3_ESM.pdf]

nature portfolio

Corresponding author(s): Amita Tiyaboonchai

Last updated by author(s): Oct 31, 2022

Reporting Summary

Nature Portfolio wishes to improve the reproducibility of the work that we publish. This form provides structure for consistency and transparency in reporting. For further information on Nature Portfolio policies, see our Editorial Policies and the Editorial Policy Checklist.

Statistics

For all statistical analyses, confirm that the following items are present in the figure legend, table legend, main text, or Methods section.

n/a	Confirmed	
☐	☒	The exact sample size (n) for each experimental group/condition, given as a discrete number and unit of measurement
☐	☒	A statement on whether measurements were taken from distinct samples or whether the same sample was measured repeatedly
☐	☒	The statistical test(s) used AND whether they are one- or two-sided<br>*Only common tests should be described solely by name; describe more complex techniques in the Methods section.*
☒	☐	A description of all covariates tested
☐	☒	A description of any assumptions or corrections, such as tests of normality and adjustment for multiple comparisons
☐	☒	A full description of the statistical parameters including central tendency (e.g. means) or other basic estimates (e.g. regression coefficient) AND variation (e.g. standard deviation) or associated estimates of uncertainty (e.g. confidence intervals)
☐	☒	For null hypothesis testing, the test statistic (e.g. F, t, r) with confidence intervals, effect sizes, degrees of freedom and P value noted<br>*Give P values as exact values whenever suitable.*
☒	☐	For Bayesian analysis, information on the choice of priors and Markov chain Monte Carlo settings
☒	☐	For hierarchical and complex designs, identification of the appropriate level for tests and full reporting of outcomes
☒	☐	Estimates of effect sizes (e.g. Cohen's d, Pearson's r), indicating how they were calculated

Our web collection on statistics for biologists contains articles on many of the points above.

Software and code

Policy information about availability of computer code

Data collection	FACS Diva (version 8.0.2 and 9.0); Lightcycler 96 SW (version 1.1, Roche); Gen5 (version 3.03) ; Zeiss Zen Software (ver 2.3 Blue Edition, Carl Zeiss AG); ChemiDoc Touch Imaging System (BioRad); IVIS Lumina II
Data analysis	TIDE web tool (https://tide.nki.nl/); GraphPad Prism 9.4.0 ; FlowJo 10.6.1; ImageJ 1.52k; SnapGene 6.1; Microsoft Excel (v16.64); HiSeq Control Software (version HD 3.5.0.7); Illumina bcl2fastq (ver 2.17); Bowtie (version 1); Kallisto (ver 0.44.0) ; trim-galore (ver 0.6.3); Bioconda package bioconductor-tximport (ver 1.12.1); FastQC (ver 0.11.8) and MultiQC (ver 1.7).<br>The custom code used in this study has been deposited in the Zenodo repository with DOI: 10.5281/zenodo.7268404 [https://zenodo.org/record/7268404#.Y2AQH-zMK-o].

For manuscripts utilizing custom algorithms or software that are central to the research but not yet described in published literature, software must be made available to editors and reviewers. We strongly encourage code deposition in a community repository (e.g. GitHub). See the Nature Portfolio guidelines for submitting code & software for further information.

Data

Policy information about availability of data

All manuscripts must include a data availability statement. This statement should provide the following information, where applicable:

- Accession codes, unique identifiers, or web links for publicly available datasets
- A description of any restrictions on data availability
- For clinical datasets or third party data, please ensure that the statement adheres to our policy

The authors declare that all data supporting the finding of this study are included in this published article, supplemental materials, the Source Data file. Source data are provided with this paper. The reference mouse genome assembly used is GRCm38 and annotation was made by using gencode version 24. The RNA sequencing data generated in this study have been deposited in the Gene Expression Omnibus database under the accession code GSE216550 [https://www.ncbi.nlm.nih.gov/geo/query/acc.cgi?acc=GSE216550].

Human research participants

Policy information about studies involving human research participants and Sex and Gender in Research.

Reporting on sex and gender	not applicable.
Population characteristics	not applicable.
Recruitment	not applicable.
Ethics oversight	not applicable.

Note that full information on the approval of the study protocol must also be provided in the manuscript.

Field-specific reporting

Please select the one below that is the best fit for your research. If you are not sure, read the appropriate sections before making your selection.

- [x] Life sciences
- [ ] Behavioural & social sciences
- [ ] Ecological, evolutionary & environmental sciences

For a reference copy of the document with all sections, see nature.com/documents/nr-reporting-summary-flat.pdf

Life sciences study design

All studies must disclose on these points even when the disclosure is negative.

Sample size	No statistical test were used to determine sample size ahead of time. The required sample size was estimated based on preliminary data taking into consideration experimental approach, feasibility and availability.
Data exclusions	6 of 76 mice where facial vein injection did not go into the vein and resulted in a visible subcutaneous bleb on the mouse face were excluded. 4 of 87 mice that had failed liver perfusion due to human technical error were also excluded as single hepatocytes could not be isolated from these livers and as a result analytical flow cytometry could not be run on the samples.
Replication	All experiments were repeated a minimum of 2 times with at least a group of 4 mice each with mice born from different breeding pairs. All experiments were successfully reproduced.
Randomization	Mice were randomly designated to control and experimental groups.
Blinding	The researcher was blinded as to whether the mice received either saline, GR-Cypor scgRNA, or GR-Cypor scgRNA during APAP injection for selection. The investigator was not blinded in other experiments as the majority of experiments were performed by a single investigator. The collected data were not observational, but quantitative and so would not be influenced by subjective researcher bias.

Reporting for specific materials, systems and methods

We require information from authors about some types of materials, experimental systems and methods used in many studies. Here, indicate whether each material, system or method listed is relevant to your study. If you are not sure if a list item applies to your research, read the appropriate section before selecting a response.

Materials & experimental systems

n/a	Involved in the study
	☒ Antibodies
☒	☐ Eukaryotic cell lines
☒	☐ Palaeontology and archaeology
	☒ Animals and other organisms
☒	☐ Clinical data
☒	☐ Dual use research of concern

Methods

n/a	Involved in the study
☒	☐ ChIP-seq
	☒ Flow cytometry
☒	☐ MRI-based neuroimaging

Antibodies

Antibodies used	rabbit anti-Cypor (Abcam, catalog #180597, dilution 1:200); goat-anti-human F9 (Affinity Biologicals, catalog # GAFIX-AP, dilution 1:100); Alexa-Fluor 647 donkey-anti-rabbit IgG (Jackson ImmunoResearch catalog #711-606-152, dilution 1:400); Cy3 donkey-anti-goat IgG (Jackson ImmunoResearch, catalog #705-165-147, dilution 1:400); rabbit-anti-mouse Albumin (Cell Signaling, catalog#4929, diluted 1:1000); rabbit-anti-Gapdh 14C10 (Cell Signaling, catalog#2118, diluted 1:1000); Donkey-anti-mouse IgG-HRP(Prometheus protein biology products, catalog# 20-304D, diuted 1:10,000).
Validation	Commercially available antibodies were validated by their respective vendors as follows:<br>https://www.abcam.com/cytochrome-p450-reductase-antibody-epr14479b-ab180597.html<br>https://affinitybiologicals.com/product/factor-ix-polyclonal-antibody-affinity-purified-goat/<br>https://www.jacksonimmuno.com/catalog/products/711-606-152<br>https://www.jacksonimmuno.com/catalog/products/705-165-147<br>https://www.cellsignal.com/products/primary-antibodies/albumin-antibody/4929<br>https://www.cellsignal.com/products/primary-antibodies/gapdh-14c10-rabbit-mab/2118<br>https://geneseesci.com/shop-online/product-details/20-304D/prometheus-protein-biology-products-20-304d-donkey-anti-mouse-igg-h-l-hrp-linked-whole-ab-secondary-antibody-1mg-ml-500ul-unit?search=Donkey%20anti%20mouse

Animals and other research organisms

Policy information about studies involving animals; ARRIVE guidelines recommended for reporting animal research, and Sex and Gender in Research

Laboratory animals	All animals were fed tap water and standard mouse chow (LabDiet Picolab Rodent Diet 5LOD). Animals were housed under a standard 12-hour on and off light cycle in groups of 2 to 5 animals in the Department of Comparitive Medicine at the Oregon Health and Science University. The animal room was maintained at an ambient temperature of 70 degrees Farenheit and 30 %-70% humidity.<br>Wild type C57BL/6 , B6J-Rosa-Cag-spCas9 (Stock #026179) and B6-Rosa-Cas9-Ai9tdTomato (Stock#007909) mice were obtained from Jackson Laboratories. Experimental mice ranged in age between neonates up to 12 weeks old dependent on the experiments.<br>Fah-/- mice were of the Fah with deletion of exon5 strain on the 129s4 background and were maintained on drinking water containing 8mg/L NTBC. In the experiments, mice received the rAAV as neonates.
Wild animals	The study did not involve wild animals
Reporting on sex	Mice of both sex were randomly assigned to control and experimental groups.
Field-collected samples	The study did not involve samples collected from the field.
Ethics oversight	All animal experiments were approved by the Institutional Animal Care and Use Committee (IACUC) at Oregon Health & Science University.

Note that full information on the approval of the study protocol must also be provided in the manuscript.

Flow Cytometry

Plots

Confirm that:

☒ The axis labels state the marker and fluorochrome used (e.g. CD4-FITC).

☒ The axis scales are clearly visible. Include numbers along axes only for bottom left plot of group (a 'group' is an analysis of identical markers).

☒ All plots are contour plots with outliers or pseudocolor plots.

☒ A numerical value for number of cells or percentage (with statistics) is provided.

Methodology

Sample preparation	Hepatoctyes were isolated by a two-step collagenase perfusion of the liver. Hepatocytes were then collected by sequential centrifugation at 50 x g for 5 minutes.
Instrument	BD Symphony and BD Fortessa
Software	FACS Diva and FlowJo V10.6
Cell population abundance	No cell sorting was performed, only analytical flow cytometry.
Gating strategy	Non-cell events were excluded by gating by using SSC-A vs FSC-A. Doublets and larger cell clusters were then excluded by gating with FSC-A vs FSC-W. TdTomato+ hepatocytes were gated based on a negative control (either a mouse that received a saline injection or no treatment) where axis were set to 561 vs 488.

☒ Tick this box to confirm that a figure exemplifying the gating strategy is provided in the Supplementary Information.
